# Investigating the influence of varied ratios of red and far-red light on lettuce (*Lactuca sativa*): effects on growth, photosynthetic characteristics and chlorophyll fluorescence

**DOI:** 10.3389/fpls.2024.1430241

**Published:** 2024-09-10

**Authors:** Xueting Bi, Hong Xu, Chaowei Yang, Haoran Zhang, Wei Li, Wei Su, Mingtao Zheng, Bingfu Lei

**Affiliations:** ^1^ Key laboratory for Biobased Materials and Energy of Ministry of Education, College Materials and Energy, South China Agricultural University, Guangzhou, China; ^2^ College of Horticulture, South China Agricultural University, Guangzhou, China; ^3^ Maoming Branch, Guangdong Laboratory for Lingnan Modern Agriculture, Maoming, China

**Keywords:** far-red light, phosphor converted LED, lettuce, stomata, photosynthetic characteristics

## Abstract

Far red photon flux accelerates photosynthetic electron transfer rates through photosynthetic pigments, influencing various biological processes. In this study, we investigated the impact of differing red and far-red light ratios on plant growth using LED lamps with different wavelengths and Ca_1.8_Mg_1.2_Al_2_Ge_3_O_12_:0.03Cr^3+^ phosphor materials. The control group (CK) consisted of a plant growth special lamp with 450 nm blue light + 650 nm red light. Four treatments were established: F1 (650 nm red light), F2 (CK + 730 nm far-red light in a 3:2 ratio), F3 (650 nm red light + 730 nm far-red light in a 3:2 ratio), and F4 (CK + phosphor-converted far-red LED in a 3:2 ratio). The study assessed changes in red and far-red light ratios and their impact on the growth morphology, photosynthetic characteristics, fluorescence characteristics, stomatal status, and nutritional quality of cream lettuce. The results revealed that the F3 light treatment exhibited superior growth characteristics and quality compared to the CK treatment. Notably, leaf area, aboveground fresh weight, vitamin C content, and total soluble sugar significantly increased. Additionally, the addition of far-red light resulted in an increase in stomatal density and size, and the F3 treatments were accompanied by increases in net photosynthetic rate (Pn), transpiration rate (Tr), intercellular CO_2_ concentration (Ci), and stomatal conductance (Gs). The results demonstrated that the F3 treatment, with its optimal red-to-far-red light ratio, promoted plant growth and photosynthetic characteristics. This indicates its suitability for supplementing artificial light sources in plant factories and greenhouses.

## 1 Introduction

In recent years, facility gardening has gained attention for enhancing horticultural productivity (Van Gerrewey et al., 2022), offering higher and more predictable yields per unit area while optimizing resource usage (Van Delden et al., 2021). Greenhouses, a popular choice for protected cultivation, often necessitate artificial lighting with specific intensity and spectral composition. The increasing use of energy-efficient LEDs in protected cultivation systems has rekindled interest in understanding light quality’s impact on crop productivity (Wassenaar et al., 2022).

Photosynthesis is the process through which plants utilize light energy to convert carbon dioxide (CO_2_) and water (H_2_O) into organic matter, releasing oxygen (O_2_) in the process (Wang et al., 2020). Light quality significantly influences photosynthesis (Parys et al., 2021). Given that light in the 400-700 nm wavelength range is most efficient for photosynthesis (Bautista-Saraiva et al., 2018), many studies have focused on this range (Matsuda et al., 2004; Baba et al., 2012). White light, comprising integrated wavelengths, is recognized as crucial for promoting normal plant growth as it provides ample energy for photosynthesis. However, recent research highlights the significance of red light in plant growth. Red light (600-700 nm), commonly used in plant factories with artificial lighting (PFAL), has been found to enhance biomass, leaf area, leaf length, leaf height, and soluble sugars, while reducing nitrate levels in green leafy vegetables (Baba et al., 2012).

Far-red light supplementation, which is adding far-red light to white light in combined light qualities, significantly enhances quantum yield and net photosynthesis of photosystem II while reducing non-photochemical fluorescence quenching (Zou et al., 2019; Zhen and Bugbee, 2020) This supplementation results in increased light use efficiency and plant biomass. Additionally, when combined with light of shorter wavelength, far-red photons (701-750 nm) have been demonstrated to drive photosynthesis as effectively as photons in the photosynthetically active radiation (PAR) region (400-700 nm) (Wong et al., 2020; Zhen et al., 2021). Moreover, far-red light exhibits a synergistic effect when combined with photons in the 400-700 nm range, enhancing the efficiency of PS II in lettuce. This was observed by Emerson (Emerson et al., 1957) and confirmed by recent studies (Zhen and van Iersel, 2017; Zhen et al., 2021). Zhen demonstrated that supplementing 110 μmol m^-2^·s^-1^ of far-red light (700-770 nm) with increasing intensities of red and blue or white light (ranging from 50-750 μmol m^-2^·s^-1^) enhances photochemical efficiency and carbohydrate synthesis. Far-red light preferentially excites photosystem I, which tends to be under-excited without it, thus restoring the balance between the two photosystems and ultimately improving overall photosynthetic efficiency (Zhen and Bugbee, 2020).

Far-red light (701-750 nm) can modulate plant morphology, including adjustments to leaf angle, increased plant height, and expanded leaf area to optimize light absorption and boost crop biomass (Park and Runkle, 2018; Tan et al., 2022). Far-red light-induced shade avoidance syndromes, including promoted shoot elongation and enlarged leaves, as documented by (Franklin and Quail, 2010; Park and Runkle, 2018; Meng et al., 2019), may facilitate better light interception and lead to a substantial biomass increase in PFAL. To investigate plant responses to various light qualities, the researchers conducted a red + far-red light study in addition to natural light. The findings from lettuce light treatments demonstrated that red light + far-red light could enhance soluble sugars in lettuce and reduce nitrate content, thereby improving lettuce quality (Chen et al., 2016). In recent years, phosphors can absorb light and re-emit it in a different color (fluorescence or phosphorescence) and these phosphors emit specific light wavelengths, providing plants with light of broad spectral distribution (Yang et al., 2023). By manipulating the combination of phosphor wavelengths, precise control over plant growth can be achieved. Phosphors absorb incident light and convert it into different wavelengths, enhancing the light received by plants. This promotes photosynthesis and efficiency (Wu et al., 2022). Different phosphor types possess varying properties regarding absorbed and emitted light wavelengths, complementing the specific spectrum needed by plants. For instance, some phosphors can increase the proportion of red or blue light, crucial for plant growth and photosynthesis. Through spectrum modulation, phosphors create a more suitable light environment for plant photosynthesis and growth (Fang et al., 2022).

Various light qualities exert unique influences on the photosynthesis and growth of plants. A thorough understanding of these mechanisms can assist in optimizing the plant growth environment and enhancing crop yields to address the escalating challenges of food security. In this study, lettuce was the research subject. We investigated the selection of different far-red light ratios and the introduction of a new luminescent material. The objective was to assess the impact of diverse light conditions on plant growth and photosynthesis by measuring growth indices (e.g., plant height, leaf area, biomass) and photosynthetic parameters (e.g., chlorophyll content, photosynthesis rate, and respiration rate). The aim of this study was to explore the effects of different ratios of red light and far-red light on plant growth and photosynthesis, and to explore the application effect of phosphor, to provide a scientific basis for the optimization of plant cultivation and photosynthesis.

## 2 Article types

Original Research

## 3 Materials and methods

### 3.1 Plant materials and experimental setup

**Figure 1:**
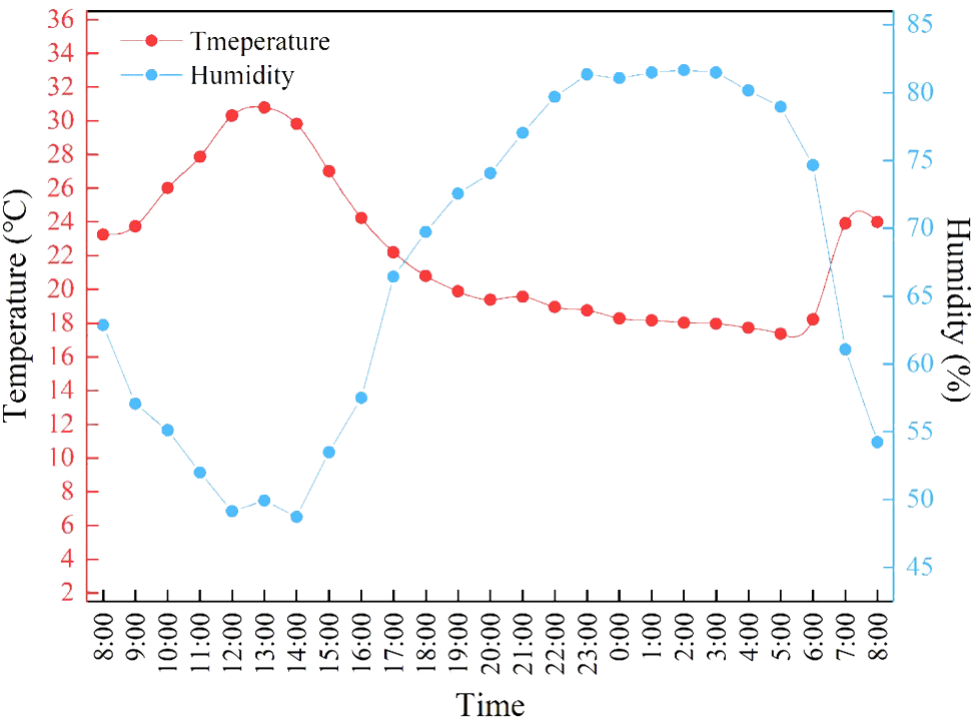
Average daily temperature and humidity in greenhouses from November to December.

The experiment spanned from November 10 to December 30, 2023, within a Venlo-type glass greenhouse at the College of Materials and Energy (South China Agricultural University, Guangzhou, China). The greenhouse climate conditions are shown in **Figure 1**. Cream Lettuce (Hebei Nanjixing Seed Co. Ltd., Guangzhou, China) was chosen as the lettuce variety (*Lactuca sativa*) for the study. The conditions for seedling cultivation are as follows: natural light conditions supplemented with LED lighting to maintain a consistent light intensity of 200 µmol/m²/s during daylight hours, temperature of 20°C ± 2°C, light cycle of 12 hours per day, and relative humidity controlled between 70% and 90%. After soaking and cleaning the lettuce seeds, they are sown on seedling sponges. When the second true leaf of the seedlings is fully expanded, uniform seedlings in terms of shape and growth are selected and transplanted into hydroponic troughs using the Deep Flow Technique (The dimensions of the DFT device:120*400*150 cm) The planting density in the DFT system was set at 30 plants per square meter. The nutrient solution prepared using the Hogland formula. The pH of the Hogland solution is adjusted to 6.0, and the electrical conductivity (EC) is set to 2 mS/cm. A supplemental light mode was employed at night, with a 12-hour photoperiod (20:00 p.m. to 8:00 a.m.), daytime average temperature at 25 ± 5°C, nighttime temperature at 18°C, and controlled relative humidity at 75 ± 5%. Obvious growth phenotypes emerged 7 days post-transplanting. Each light treatment involved planting 50 lettuce plants, replicated three times. To ensure the rigor of the experimental design, each replication was re-randomized across different plots to mitigate location-specific environmental influences. This approach ensured that no single treatment was consistently applied to the same plot, thus reducing potential biases.

### 3.2 Lighting treatment

**Figure 2:**
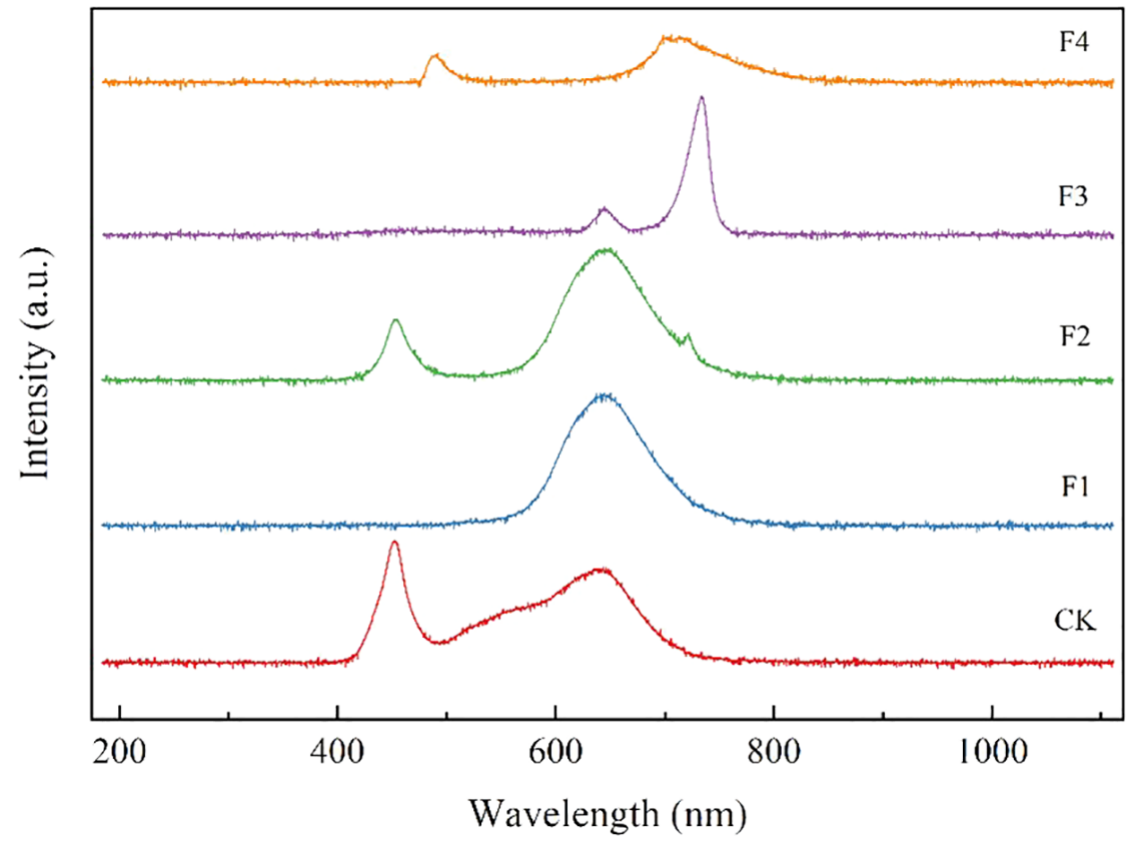
Emission spectra of different treatment.

Various LED light processing techniques were applied to the split tube from Yueqing City, Jia Cheng Lighting Co., Ltd. For the F4 processing, phosphor-converted far-red light was generated usingCa_1.8_Mg_1.2_Al_2_Ge_3_O_12_:0.03Cr^3+^ phosphor under 450 nm chipexcitation (Yang et al., 2024). The distribution of spectral settings of different treatments and the production process of excitation phosphor are shown in **Table 1**; **Figure 2**. Light quality measurements for each treatment were conducted using the Aurora4000 Series High-Resolution Spectrometer (Changchun Institute of Optics, Fine Mechanics and Physics, Chinese Academy of Sciences, Changchun, China), and light intensity measurements were conducted using the photosynthetically Active Radiation sensor (LI-190R, Lincoln, NE, USA).

**Table 1:** Different light formulations and light quantum densities.

Treatments	Light quality formulations	Light intensity [μmol/(m^2^·s)]
CK	450 nm Blue light + 650 nm Red light (1:1)	300
F1	650 nm Red light	300
F2	CK + 730 nm Far-red light (3:2)	300
F3	650 nm Red light + 730 nm Far-red light (3:2)	300
F4	CK + Phosphor Stimulated Far-red LED (3:2)	300

### 3.3 Stomata observation

After 45 days of growth, lettuce plants were sampled, and leaf slices were collected from five plants. To prepare leaf epidermal sections, 1 cm² leaf slices were uniformly coated with colorless transparent nail polish. After complete drying, the polish-coated slices were gently removed and placed on slides to create water-sealed plant slices (Li et al., 2010).

For leaf longitudinal sections, a hand sectioning method was employed. These prepared sections were then examined and photographed using the Nikon inverted fluorescence microscope imaging system (Ts2, Nikon Corporation, Japan) with a 10x eyepiece and a 40x objective. Stomatal status in the epidermal sections was observed at the same magnification.

### 3.4 Photosynthetic properties and chlorophyll fluorescence

The Li-6400XT Portable Photosynthesis Measurement System (LI-COR Biosciences, Lincoln, NE, USA) from Li-Cor was employed to assess plant photosynthetic parameters 45 days after planting. Three plants exhibiting consistent and uniform growth were selected for each treatment. Functional leaves from the third position from the bottom of the plants were chosen to determine photosynthetic parameters., including leaf net photosynthetic rate (*Pn*), transpiration rate (*Tr*), stomatal conductance (*Gs*), and intercellular CO_2_ concentration (*Ci*). Water utilization efficiency (WUE), calculated as *Pn*/*Tr*, was determined for each leaf, with the process repeated three times (The settings for the measurements were as follows: the light intensity was set to 800 µmol m^−2^ s^−1^ using the LI6800-01A light source with a light quality of 20R80B, the leaf temperature was maintained at 25°C, and the relative humidity inside the leaf chamber was kept at 70%, and the CO_2_ concentration was set to 800 ppm. The airflow rate through the chamber was set to 1000 µmol s^−1^. The leaf area used for measurements was 8 cm²).

Using the Imaging-PAM chlorophyll fluorometer (IMAGMAX1, Heinz Walz, Effeltrich, Germany), three plants were measured for each treatment, selecting the first fully expanded functional leaf for measurement. The instrument was set with a leaf chamber area of 8 cm² and a light intensity of 1000 µmol·m^−2^·s^−1^ (chlorophyll fluorescence was measured under saturation light conditions.). To construct the light response curve in photosynthetically active radiation (PAR) levels were incrementally adjusted, and measurements were taken at each level after a stabilization period of 2 minutes. The specific PAR levels used were 0, 50, 100, 200, 400, 600, 800, and 1000 µmol·m^−2^·s^−1^. Each measurement was performed with a wait time of 2 minutes between adjustments to ensure accurate readings. Measurements included the relative electron transport rate (rETR), maximum fluorescence (F’m), steady-state fluorescence (Fs), and minimum fluorescence (F’o).

The relative electron transport rate (rETR):


(1) $$\text{rETR=}\Phi_{\text{PSII}}\times \text{PPFD}$$

The effective quantum yield of PSII (ΦPSII):


(2) ΦPSII=F'm−FsF'm

Subsequently, the same leaf area was dark-adapted for 20 minutes to measure the initial fluorescence (Fo) and maximum fluorescence (Fm). Based on these chlorophyll fluorescence parameters, the variable fluorescence Fv = Fm - Fo, the maximum photochemical efficiency of photosystem II (Fv/Fm) = (Fm - Fo)/Fm, the potential photochemical efficiency of PSII (Fv/Fo) = (Fm - Fo)/Fo, the photochemical quenching coefficient (qP) = (F’m - Fs)/(F’m – F’o), and the non-photochemical quenching coefficient (NPQ) = (Fm – F’m)/F’m were calculated.

### 3.5 Growth parameters

Five plants with consistent growth under different light treatments were randomly selected for growth analysis. The leaf length and leaf width were measured using a tape measure and a vernier scale. Vernier calipers were used to measure stem thickness and petiole thickness; the number of leaves was calculated by the direct method (Use a plant marking pen to gently mark each leaf of the lettuce, and directly count each leaf); root data measurement is performed using a root scanner (WINRHIZO, Chengyi Imp & Exp Co., Ltd, Guangzhou, China). the aboveground and belowground fresh mass of lettuce was determined using an electronic balance (FA1004E, Sanlitech, China), and the aboveground and belowground dry masses of lettuce were determined using an electronic balance after lettuce was dried in an oven at 80°C for 72h to a constant mass; the strong seedling index was calculated by the formula, i.e., the strong seedling index was calculated as strong seedling index = (stem thickness/plant height + belowground dry mass/aboveground dry mass) × the whole dry mass. Mass per unit of leaf area (LMA) = dry mass/single leaf area. (Teklehaimanot, 2004).

For each treatment, 5 lettuce leaves were collected and processed with liquid nitrogen to grind into powder (stored in a -80°C freezer). The photosynthetic pigment content was determined by acetone ethanol mixing method (Wellburn, 1994). The leaf soluble protein content was determined by colorimetric method (Bradford, 1976). The leaf total soluble sugar content was determined by anthrone sulfate method (Irigoyen et al., 1992). The leaf Vitamin C content was determined by molybdenum blue colorimetric method (Omaye et al., 1979), and leaf nitrate content was measured by the salicylic acid-sulfuric acid colorimetric method (Miranda et al., 2001).

### 3.6 Statistical analysis

All the statistical analyses were performed using IBM SPSS 20.0 software (IBM SPSS Statistics, IBM Corporation, Armonk, NY, USA). Principal Component Analysis (PCA) was conducted to reduce the dimensionality of the dataset and to identify the principal components that explain the most variance. The data were first standardized to have mean zero and unit variance. The covariance matrix was then computed, and eigenvalues and eigenvectors were extracted to determine the principal components. The number of components retained was based on the eigenvalues greater than 1 criterion and a scree plot examination. Different lowercase letters represent significant differences between the treatments according to Duncan’s multiple range test (one-way ANOVA, *p*< 0.05). Means separation was determined using the Tukey-Kramer HSD method (*p* = 0.05). The figures were plotted using Origin 2021.

## 4 Results

### 4.1 Impact of different light treatments on plant growth

**Figure 3:**
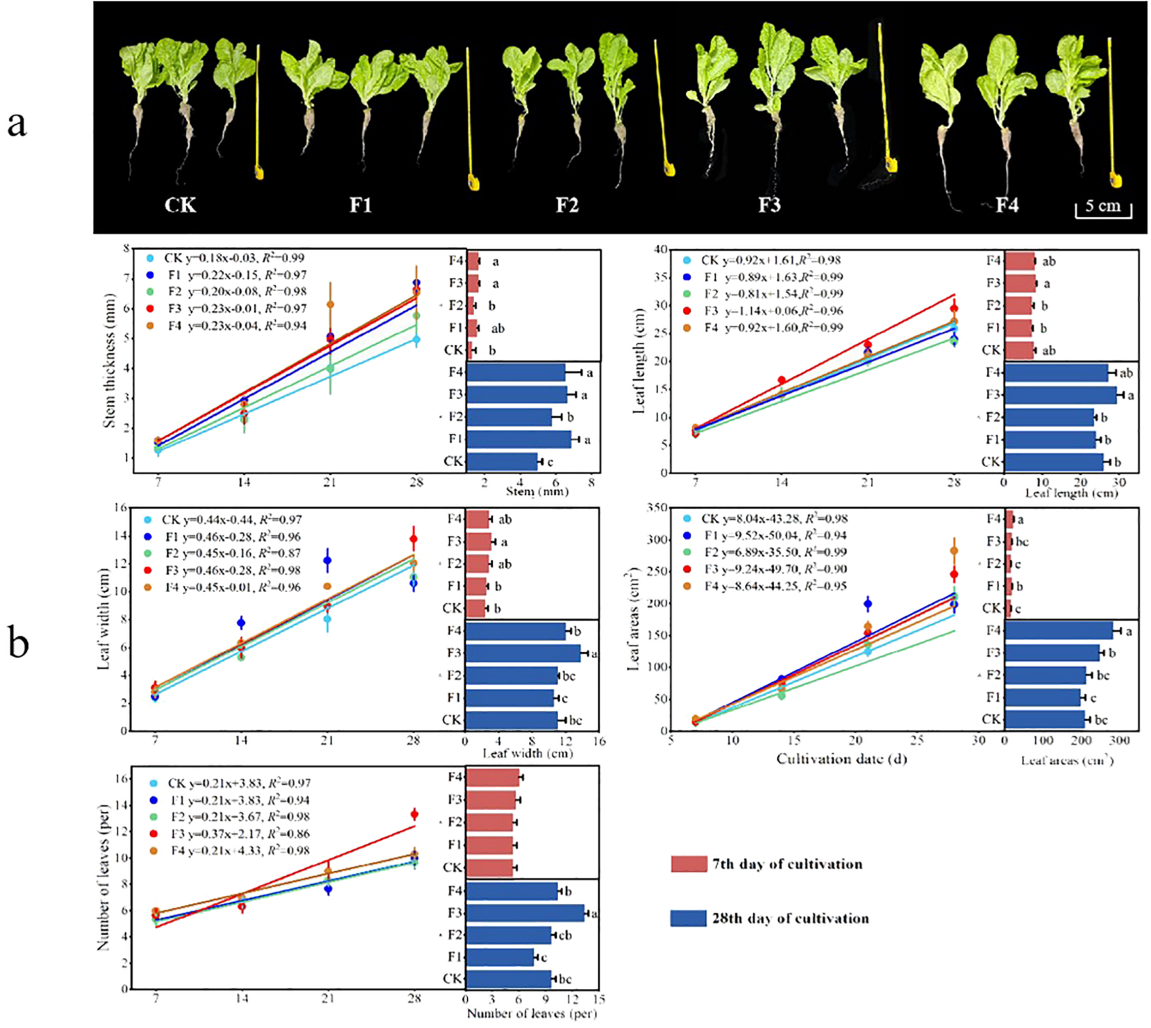
Fit curves and comparative analysis of lettuce growth during different light treatments: **(A)** Lettuce plant control, **(B)** lettuce growth fitting curve. Different lowercase letters in the same column in the Figure indicate that the difference between different treatments reaches a significant level of *p<* 0.05. The data fitting was performed using linear regression, with *R^2^* > 0.80 and *P* > 0.75, indicating a high level of fitting accuracy.

The growth indices, including stem thickness, leaf length, leaf width, total area, and the number of leaves, were measured and fitted for lettuce plants subjected to different light quality treatments (**Figure 3B**). The results indicated variations in lettuce growth among the different light quality treatments (**Figure 3A**). On the 28th day of lettuce growth, the growth indices revealed notable differences. Stem thickness for F3 increased by 27.6% and 15.1% compared to CK and F2, respectively. Leaf length exhibited increases of 13.6%, 22.9% and 25.3% compared to CK, F1 and F2, respectively. Leaf width surpassed CK, F1, F2, and F4 by 24.6%, 30.1%, 25.1%, and 14.68%, respectively. Leaf area increased by 17.3%, 24.1%, and 15.7% compared to CK, F1, and F2, and was 12.9% less than F4. The number of leaf blades saw a significant increase by 37.9%, 73.9%, 37.9%, and 29.1%. The optimal lighting condition was found to be a 3:2 ratio of CK + Phosphor Stimulated Far-red LED (3:2) (F4), effectively regulating plant growth.

### 4.2 Impact of different light qualities on the biomass and root of lettuce

**Figure 4:**
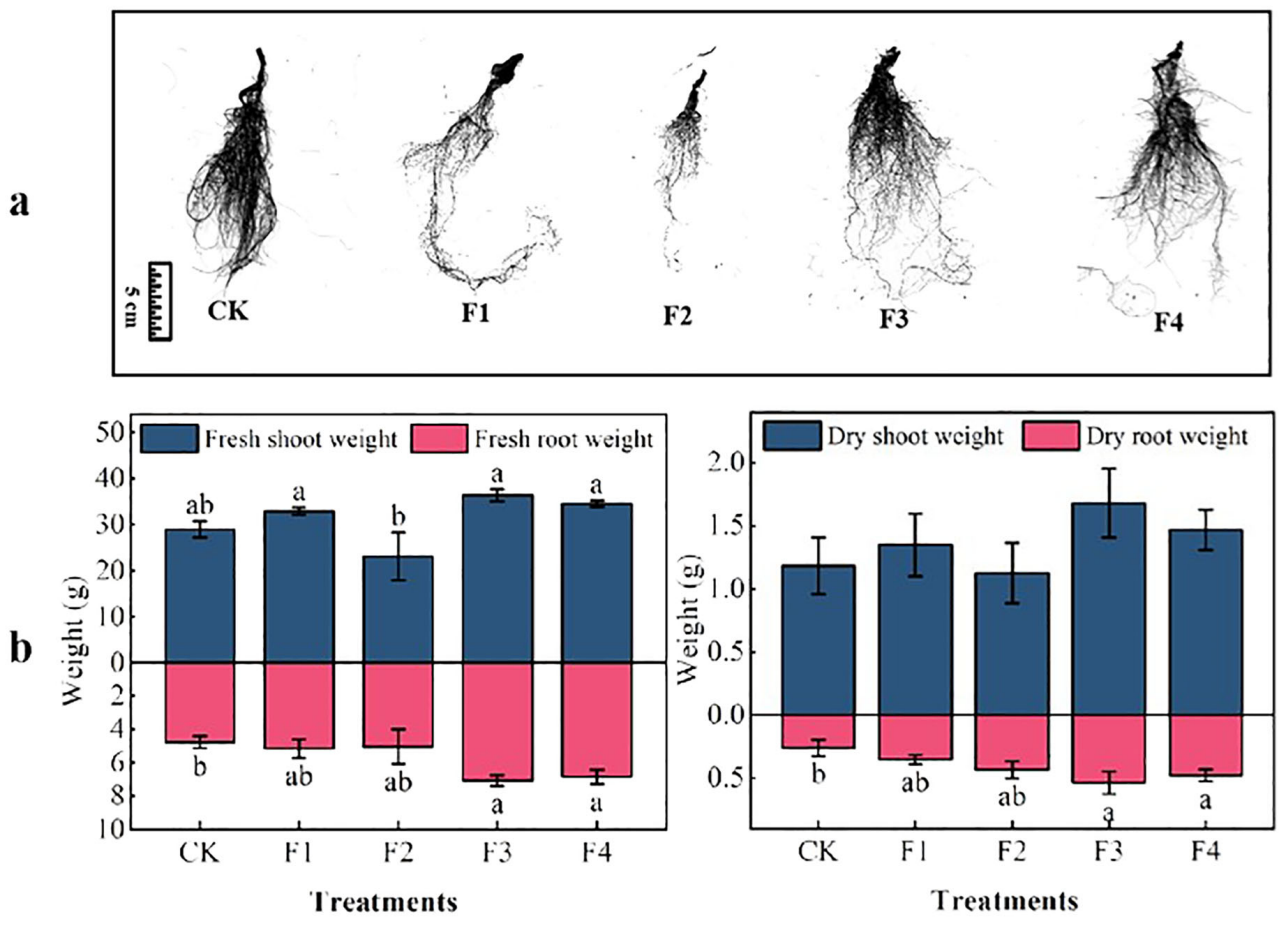
Effect of different light treatments on lettuce biomass: **(A)** root morphology, **(B)** lettuce plant biomass. Different lowercase letters in the same column in the figure indicate that the difference between different treatments reaches a significant level of *p*< 0.05.

Significant differences were observed among various light treatments concerning lettuce biomass and root, as illustrated in **Figure 4B**. Regarding aboveground fresh mass, the F3 treatment outperformed others, showing a noteworthy increase of 25.6% compared to the control (CK) and a significant difference of 57.5% compared to the F2 treatment. Concerning belowground fresh weight mass, both the F3 and F4 treatments exhibited a substantial increase of 48.3% and 43.9%, respectively, compared to the control (CK). However, the difference between F3 and F4 was not statistically significant. In terms of aboveground dry mass, direct differences among treatments were not found to be significant. However, for underground dry mass, both F3 and F4 demonstrated a significant increase of 103% and 80.7% over the control (CK), with no significant differences between F3 and F4 (**Figure 4A**).

The total root length and area of lettuce under different light treatments followed this order (**Table 2**): F3 > F1 > F4 > F2 > CK. These results suggest that optimal values for aboveground fresh mass, dry mass, belowground fresh mass, dry mass, root length, and area of lettuce were achieved under the light conditions of the F1, F3, and F4 treatments, with the F3 treatment being superior to the others. This indicates that the F1, F3, and F4 treatments significantly enhance both biomass and root length, with F3 being the most effective.

**Table 2:** Impact of varied light treatments on lettuce root development.

Treatments	Total Length (cm)	Total Surface Area (cm^2^)	Average Diameter (mm)	Root Volume (cm^3^)
CK	144.15 ± 19.71b	18.8 ± 1.03b	0.56 ± 0.05	1.24 ± 0.22ab
F1	332.92 ± 41.38a	24.54 ± 0.93a	0.49 ± 0.04	2.11 ± 0.45a
F2	206.99 ± 65.67ab	21.03 ± 1.87ab	0.41 ± 0.03	0.51 ± 0.16b
F3	347.37 ± 18.52a	24.55 ± 1.59a	0.48 ± 0.05	2.32 ± 0.33a
F4	298.37 ± 53.84a	25.16 ± 1.22a	0.5 ± 0.08	2.23 ± 0.45a

Data are means ± standard error, analysis of differences in different treatments, different letters in the same column indicate significant differences (p< 0.05).

### 4.3 Impact of different light qualities on the nutritional quality of lettuce

**Figure 5:**
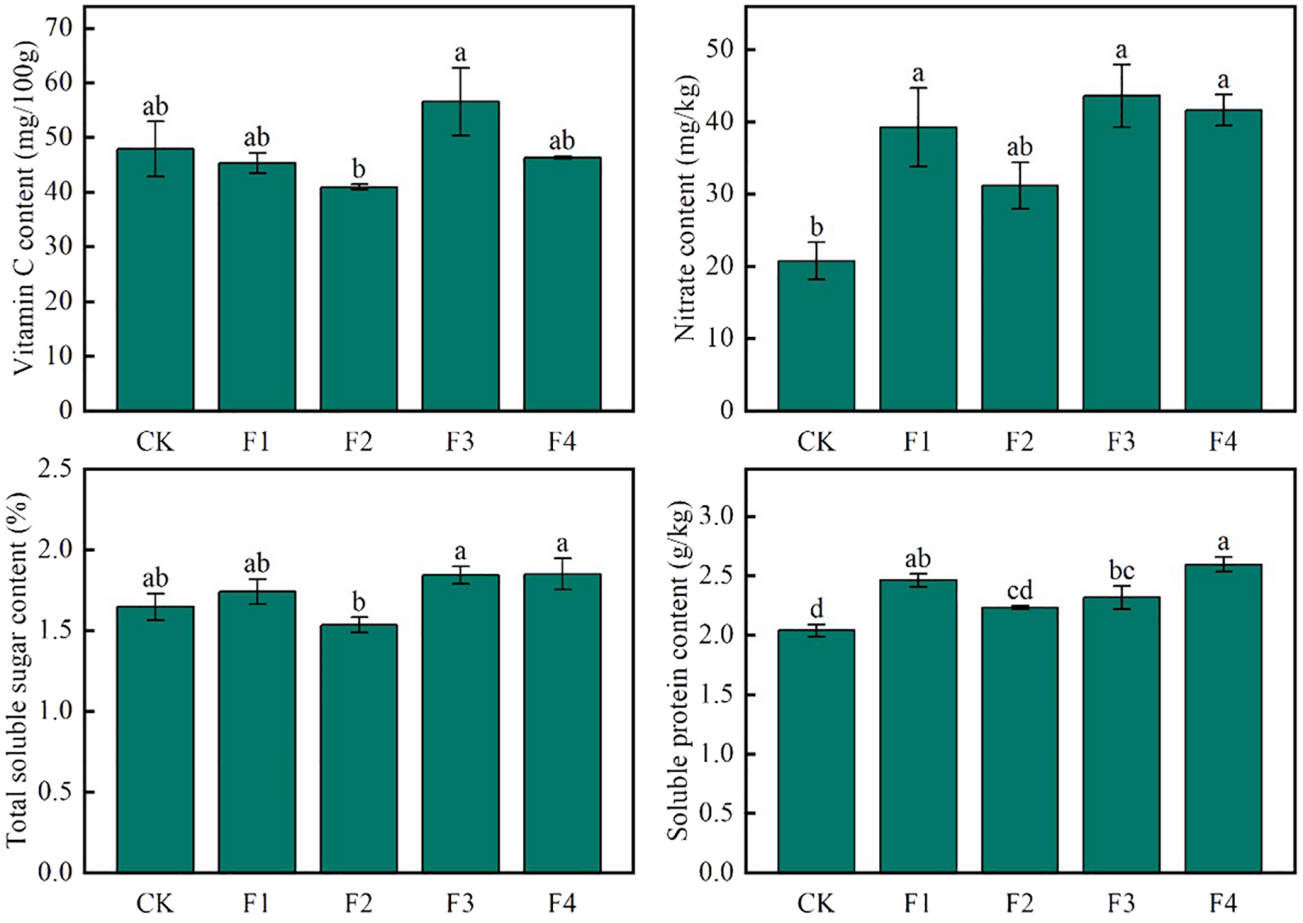
Effect of various light qualities on lettuce nutritional quality. Different lowercase letters in the same column in the figure indicate that the difference between different treatments reaches a significant level of *p*< 0.05.

**Figure 5** illustrates distinct trends in the quality indexes of lettuce leaves under various light treatments. Notably, the vitamin C content of F3 exhibited a significant increase of 38.1% compared to F2, while no significant differences were observed between CK, F1, and F4 treatments. In terms of nitrate content, F3 demonstrated a substantial increase of 110.7% compared to CK, whereas F2 showed a significant decrease of 39.8% compared to F3. No significant differences were found between F1, F3, and F4 treatments. Additionally, the soluble sugar content of both F3 and F4 significantly increased by 11.9% and 12.2%, respectively, compared to CK. However, the difference between F3 and F4 was not statistically significant, and F2 exhibited a significant decrease of 7.3% compared to CK. Furthermore, the soluble protein content of F4 displayed a significant increase of 27.5% compared to CK. These findings indicate that, among the different light treatments, F3 consistently showed higher Vitamin C content and soluble sugar content in lettuce leaf quality compared to other treatments.

### 4.4 Effects of different light treatments on stomatal structure of lettuce leaves

**Figure 6:**
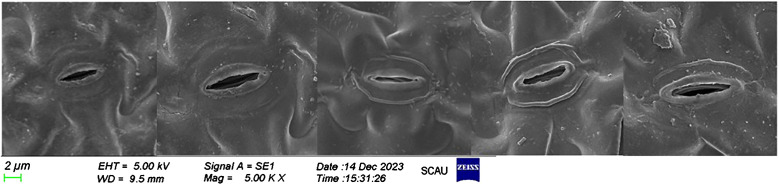
Scanning electron microscope photographs show the effects of different light treatments on the lower surface of lettuce leaves.

**Figure 7:**
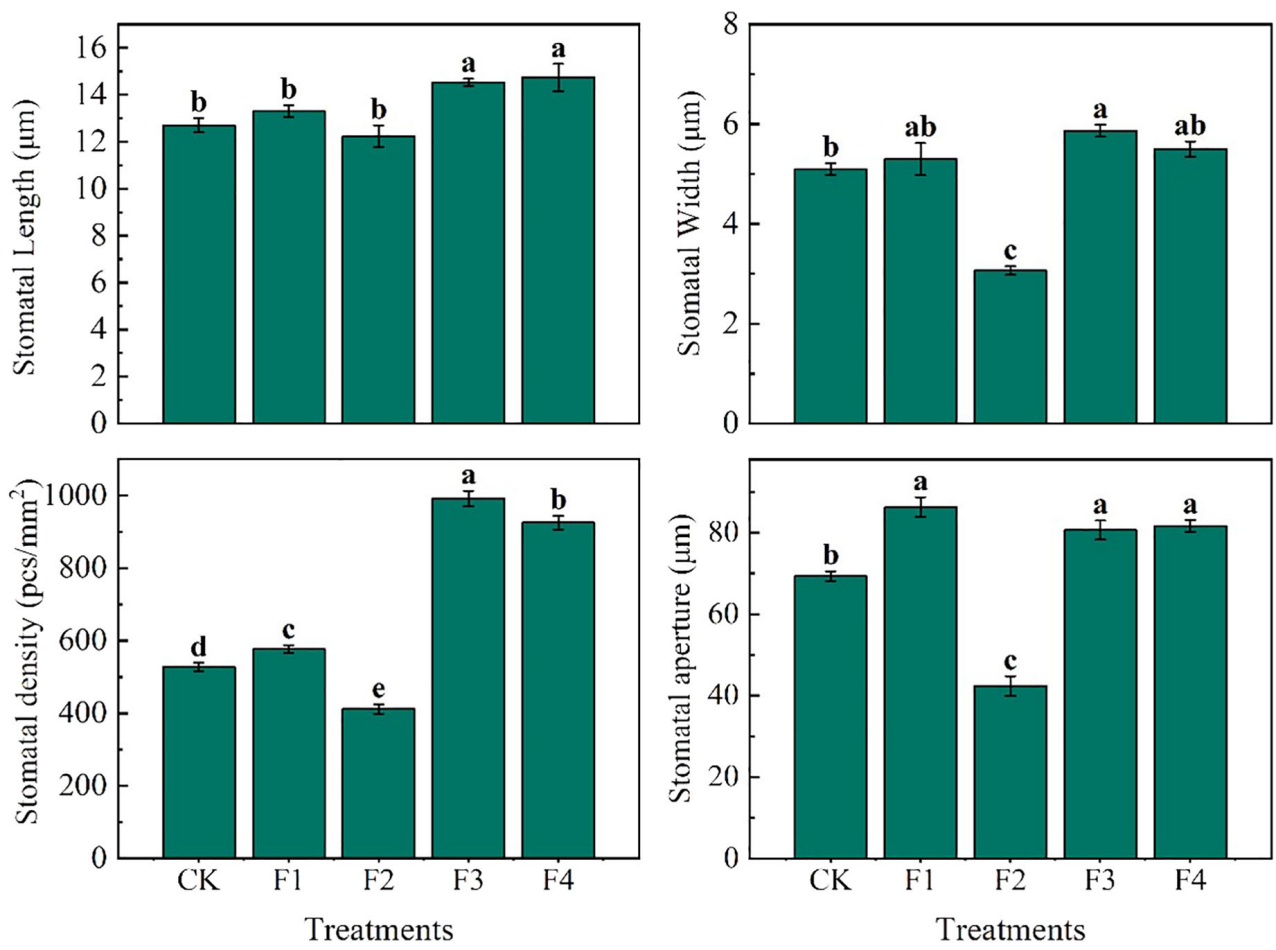
Data statistics of leaf lower-surface stomata in lettuce leaves under different light treatment. Different lowercase letters in the same column in the figure indicate that the difference between different treatments reaches a significant level of *p<* 0.05.

The experimental data reveal significant variations in the morphology and arrangement of the lower epidermis of lettuce leaves across different light treatments (**Figure 6**). Notably, compared to F2, F3 and F4 treatments exhibited the largest stomatal openings with tightly arranged stomata. The pore openings were larger, and the single area was greater in these treatments. In contrast, F2 treatment, on the other hand, featured the smallest stomatal openings, narrow and long stomata, and a small single area The individual stomatal opening of the lower-surface in lettuce leaves followed the order (**Figure 7**): F1 = F4 = F3 > CK > F2. Additionally, the order of stomatal density of the lower-surface in lettuce leaves was F3 > F4 > F1 > CK > F2.

### 4.5 Effects of different light treatments on photosynthetic pigments and photosynthetic properties of lettuce

**Figure 8:**
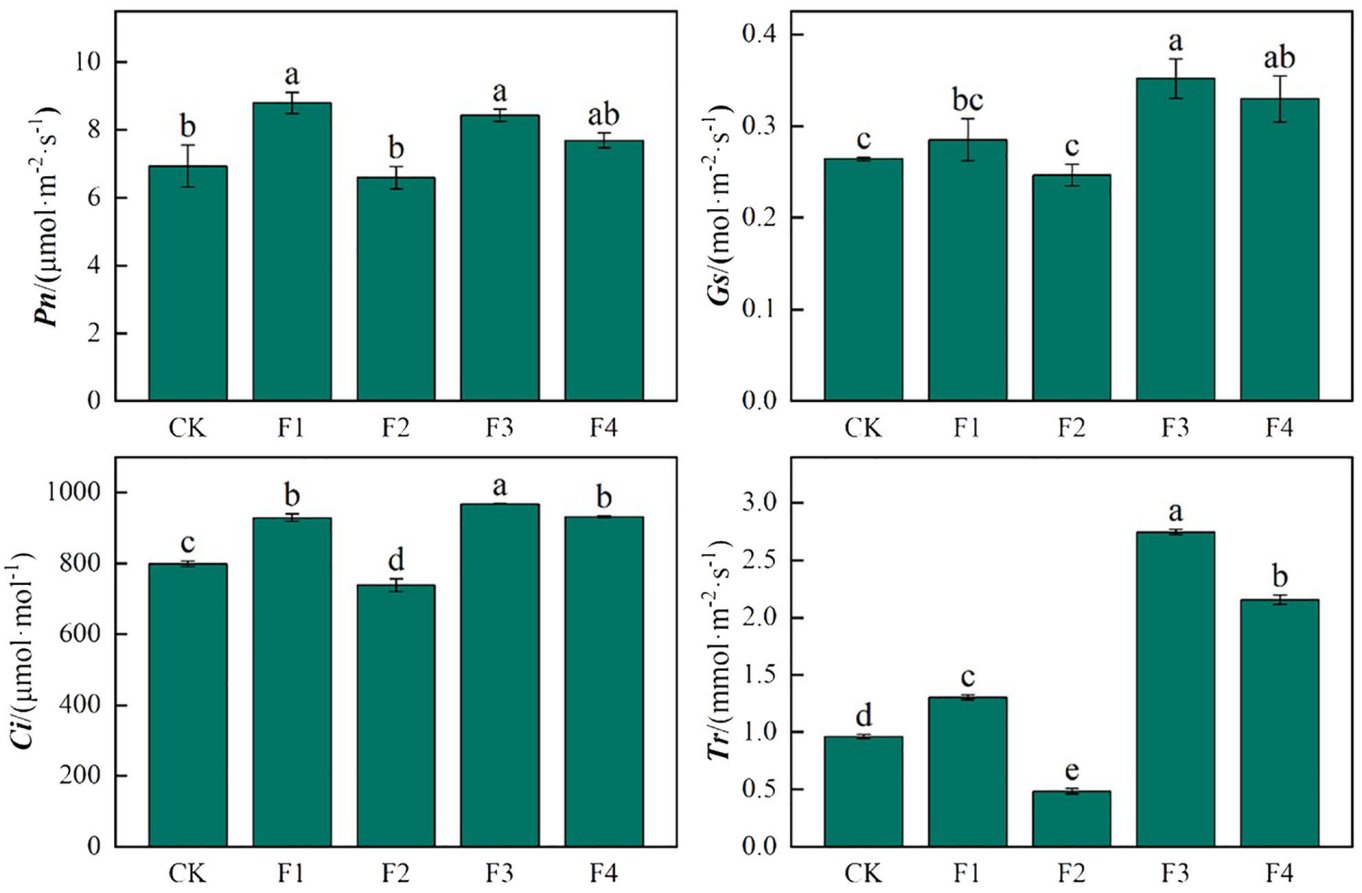
Effects of different light treatments on the photosynthetic characteristics of lettuce. Different lowercase letters in the same column in the figure indicate that the difference between different treatments reaches a significant level of *p*< 0.05.

As depicted in **Figure 8**, the photosynthetic characteristics of lettuce leaves varied significantly under different light treatments. The photosynthetic rate reached its peak in the F1 treatment, significantly surpassing that of other light treatments. Specifically, F1 However, the difference between F2 and CK was not significant. The trend of stomatal conductance (*Gs*) in lettuce leaves mirrored that of photosynthesis (*Pn*), with F3 and F4 experiencing significant increases of 32.9% and 24.6%, respectively, compared to CK. The Ci revealed that F3 and F4 exhibited significant increases of 21.2% and 16.6% compared to CK, with a significant difference between F3 and F4, while F2 decreased significantly by 8.1% compared to CK. In terms of transpiration rate (*Tr*) in lettuce leaves, there were significant differences among treatments. F1, F3, and F4 demonstrated significant increases of 35.4%, 185.4%, and 123.9%, respectively, compared to CK, whereas F2 exhibited a significant decrease of 98.3% compared to CK.

Different light treatments exerted a noteworthy impact on the pigment content of lettuce leaves (**Table 3**). The effects of all light treatments on the chlorophyll a content in lettuce leaves were not significant. Light treatments F1, F2, and F3 increased the chlorophyll b content. Among these, the F4 light treatment resulted in the highest chlorophyll b content, with increases of 31.5% and 32.4% compared to CK and F3, respectively. Additionally, light treatments F1, F2, and F3 also increased the total chlorophyll (a+b) content, with F1 having the highest total chlorophyll (a+b) content, significantly increasing by 8.7% and 6.0% compared to CK and F3, respectively. The F3 treatment had the highest chlorophyll a/b ratio, significantly increasing by 29.1%, 31.9%, and 32.2% compared to F1, F2, and F4, respectively. Compared to CK, although F1, F2, F3, and F4 light treatments increased the carotenoid content to varying degrees, only F1 and F3 showed significant differences in carotenoid content compared to CK.

**Table 3:** Effects of different light treatments on photosynthetic pigments in lettuce (mg/L).

Treatments	Chlorophyll a content	Chlorophyll b content	Chlorophyll a+b content	Chlorophyll a/b	Carotenoid
CK	15.47 ± 1.21	3.01 ± 0.56b	18.43 ± 0.13b	5.22 ± 0.79a	3.21 ± 0.41b
F1	16.04 ± 0.41	3.92 ± 0.2a	19.95 ± 0.22a	4.13 ± 0.31b	3.96 ± 0.83a
F2	15.76 ± 0.54	3.93 ± 0.18a	19.68 ± 0.37a	4.04 ± 0.33b	3.4 ± 0.68ab
F3	15.82 ± 0.11	2.99 ± 0.16b	18.81 ± 0.16b	5.33 ± 0.31a	3.67 ± 0.03a
F4	15.82 ± 0.24	3.96 ± 0.23a	19.79 ± 0.1a	4.03 ± 0.3b	3.55 ± 0.03ab

Data are means ± standard error, analysis of differences in different treatments, different letters in the same column indicate significant differences (p< 0.05).

### 4.6 Effect of different light treatments on chlorophyll fluorescence parameters

**Figure 9:**
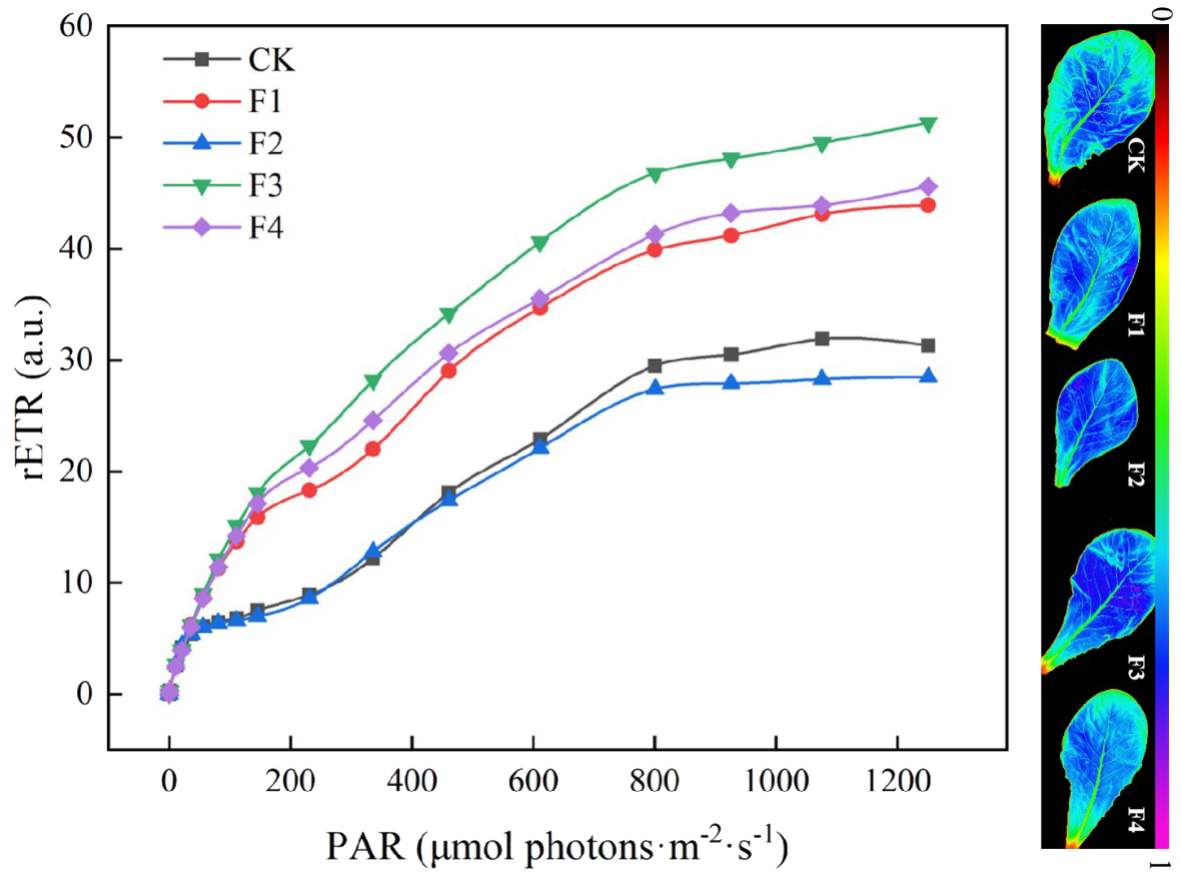
Effect of different light treatments on chlorophyll fluorescence parameters.

**Figure 9** illustrates that the relative electron transport rate (rETR), derived from Equation 1, under the F3 treatment surpasses that of other treatments. This increase in rETR is beneficial for enhancing photosynthetic efficiency and CO_2_ fixation efficiency. Additionally, the rETR of lettuce in the F3 treatment rises concomitantly with the enhancement of photosynthetic capacity.

The chlorophyll fluorescence parameters of lettuce leaves were measured under each treatment, and the corresponding data are presented in **Table 4**. Specifically, the Fv/Fm values of the F3 and F4 treatments decreased by 6.25% and 6.38% compared to the control (CK), respectively. Notably, there were no significant differences among the treatments, except for CK. The non-photochemical quenching coefficients (NPQ) for the F3 and F4 treatments were 11.9% and 3.3% lower than those of CK. Conversely, the actual photochemical quantum yield (ΦPSII), derived from Equation 2, displayed an opposite trend to Fv/Fm, with the order being F1>F3>F4>F2>CK.

**Table 4:** Effects of different light treatments on chlorophyll fluorescence parameters in lettuce leaves.

Treatments	Fv/Fm	ΦPSII	qL	qP	NPQ
CK	0.833 ± 0.005a	0.069 ± 0.033c	0.183 ± 0.08a	0.129 ± 0.036b	0.93 ± 0.033ab
F1	0.779 ± 0.012b	0.303 ± 0.039a	0.056 ± 0.017b	0.46 ± 0.051a	0.789 ± 0.073b
F2	0.804 ± 0.008b	0.078 ± 0.022c	0.227 ± 0.029a	0.172 ± 0.042b	0.959 ± 0.04a
F3	0.784 ± 0.016b	0.198 ± 0.086ab	0.053 ± 0.026b	0.338 ± 0.137a	0.831 ± 0.026ab
F4	0.783 ± 0.017b	0.097 ± 0.024bc	0.083 ± 0.022b	0.118 ± 0.056b	0.9 ± 0.04ab

Fv/Fm is the maximum photometric quantum efficiency of PSII; ΦPSII is the actual photochemical quantum yield of PSII; qL and qP is photochemical quenching; NPQ is non-photochemical quenching. Data are means ± standard error, analysis of differences in different treatments, different letters in the same column indicate significant differences (p< 0.05).

### 4.7 Photosynthetic characteristics, chlorophyll heat chart signs and growth correlation analysis of lettuce leaves

**Figure 10:**
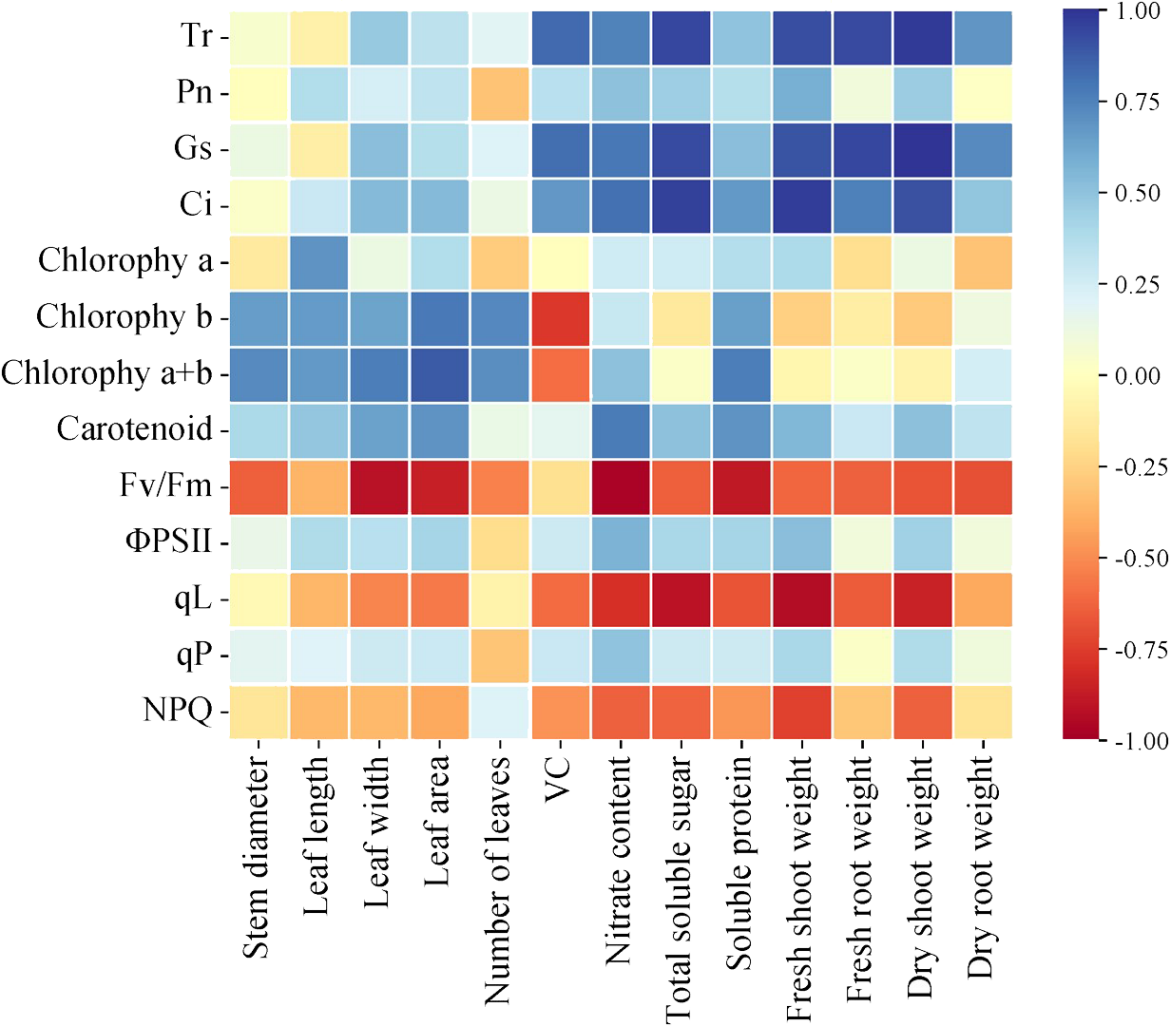
Correlation between photosynthetic characteristics and chlorophyll fluorescence components and growth parameters of lettuce leaves under different light treatments.

To comprehensively explore the interrelation among photosynthetic properties, chlorophyll fluorescence, and growth indicators in lettuce leaves and Pearson correlation heat map (**Figure 10**) were performed. Pearson correlation heat map analysis indicated significant positive correlations between *Tr*, *Pn*, *Gs*, *Ci*, and aboveground and belowground fresh weight, dry mass, ksoluble sugar, vitamin C content, nitrate content, and soluble protein content. This suggests that improved photosynthetic indexes positively influenced lettuce growth morphology and quality indices. Conversely, Fv/Fm, qL, and NPQ exhibited negative correlations with growth, quality, and biomass, emphasizing that chlorophyll content alone cannot entirely determine photosynthetic capacity, directly impacting growth and quality traits. The complex correlations among the measured photosynthetic quality indicators highlight diverse information interactions and overlaps. Singular indicators cannot serve as exclusive influencing factors for evaluating lettuce growth and quality in each treatment. Therefore, for a comprehensive assessment, the shortcomings of single indexes should be overcome. Utilizing principal component analysis, it becomes necessary to consider multiple growth and quality indicators to thoroughly evaluate lettuce growth and quality across different treatments.

### 4.8 Scores and evaluation analysis of comprehensive indicators of different light treatments

**Figure 11:**
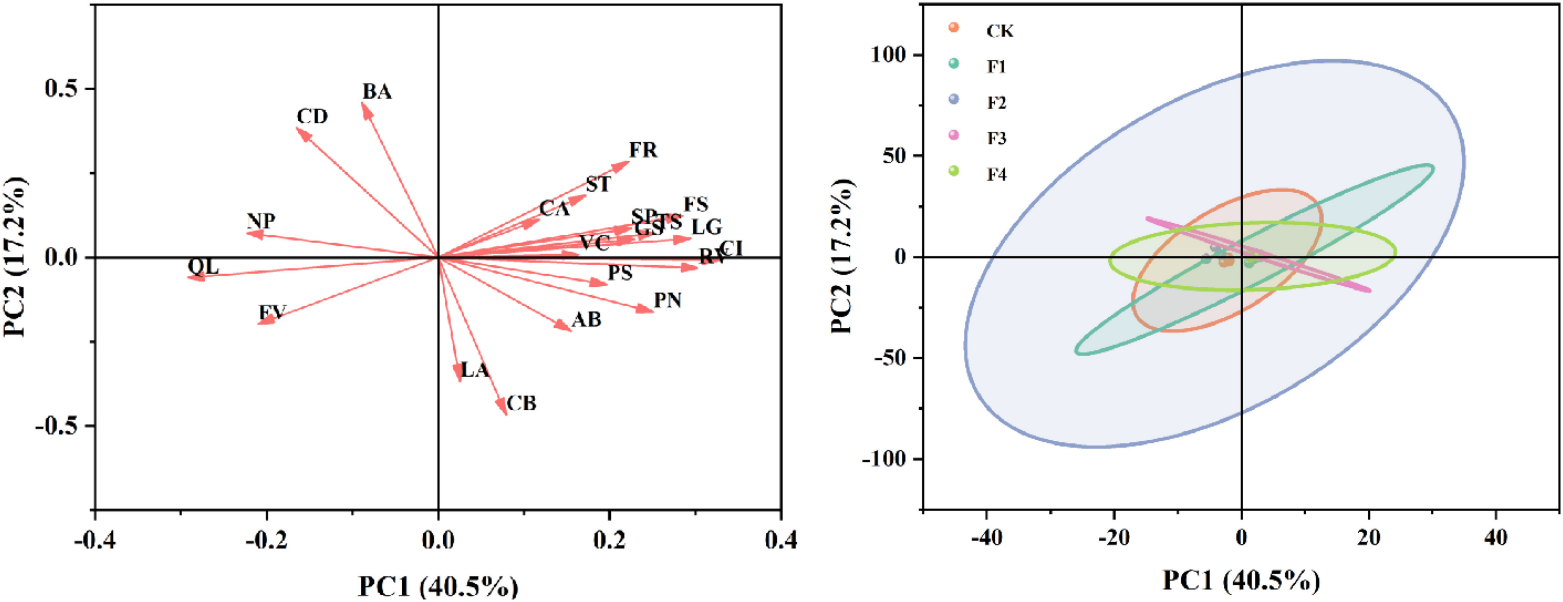
Principal component analysis (PCA) of the relationships among growth quality, biomass, photosynthetic indexes, and chlorophyll fluorescence. The direction and length of arrows indicate the correlations and their strengths, respectively. ST, stem thickness; LA, leaf area; FS, fresh shoot weight; FR, fresh root weight; VC, vitamin C; TS, total soluble sugar; SP, soluble protein; PN, photosynthesis; GS, stomatal conductance; CI, intercellular CO_2_ concentration; CA, chlorophyll a; CB, chlorophyll b; AB, chlorophyll a+b; BA, chlorophyII a/b; CD, carotenoid; LG, root length; RV, root volume; FV, Fv/Fm; PS, ΦPSII; QL, Ql; NP, NPQ.

Evident separations among the treatments were observed after conducting a principal component analysis (PCA) of growth quality, biomass, photosynthetic indexes, and chlorophyll fluorescence (**Figure 11**). PC1 explained 40.5% of the variability, highlighting its substantial role in differentiating the main trends within the data. Conversely, PC2 accounted for an additional 17.2% of the variation, although its impact was less pronounced compared to PC1. In the variable loading plot, attributes such as chlorophyll a+b (BA), chlorophyll a (CA), and stem thickness (ST) demonstrated strong positive loadings on PC1, suggesting these variables are crucial in defining the primary distinctions across the dataset. Their minimal association with PC2 implies a lesser influence on the variation explained by this component.

The sample score plot revealed clear distinctions among the treatment groups, identified by different colors (CK, F1, F2, F3, F4). The CK and F1 were significantly separated along the PC1 axis, indicating pronounced differences in principal variables between these groups. In contrast, the F3 and F4 were clustered more closely, suggesting these treatments shared similarities in the variables considered. Overall, this PCA effectively highlighted the influential roles of principal variables across different experimental treatments, delineating clear distinctions among the groups. By uncovering the most significant sources of variation, the analysis offers a detailed insight into how different conditions affect the dataset, providing a valuable foundation for further exploration of treatment effects.

By employing principal component analysis and calculating the scores for each index (**Table 5**), the comprehensive evaluation D value was derived from the membership functions u(X1), u(X2), u(X3), u(X4), and u(X5), in conjunction with weight processing. The results were ranked and presented in **Table 5**. Following a thorough analysis of comprehensive performance, considering growth quality, biomass, photosynthetic index, and chlorophyll fluorescence, the F3 treatment demonstrated the highest comprehensive evaluation D value, followed by F4 and F1. In contrast, the D values for F2 and CK treatments were the lowest. This discrepancy in evaluations could be attributed to the lower root index, chlorophyll content, and overall quality of lettuce in these treatments.

**Table 5:** Principal components, membership functions, comprehensive evaluation values(D) and rankings for different treatments.

Treatments	F1	F2	F3	F4	F5	u (X_1_)	u (X_2_)	u (X_3_)	u (X_4_)	u (X_5_)	D value	Sort
CK	-55.66	-12.25	1.06	2.45	-1.19	0.40	0.17	0.62	0.91	0.34	1.64	5
F1	50.84	-8.17	1.99	-4.06	0.38	0.92	0.28	0.62	0.40	0.73	1.89	3
F2	-105.22	10.50	-0.23	-1.20	0.51	0.11	0.60	0.35	0.49	0.61	1.77	4
F3	43.57	2.05	-0.29	2.33	1.94	0.82	0.40	0.45	0.82	0.91	2.48	1
F4	66.47	7.86	-2.53	0.47	-1.65	0.95	0.53	0.41	0.70	0.25	2.19	2

## 5 Discussion

Light, acting as both a signal and energy source for plant growth, plays a crucial role in regulating various aspects of plant development, morphogenesis, and physiological quality. Plants possess photoreceptors, known as phytochromes, that sense both red and far-red light, consisting of chromophores and apoproteins. In this experiment, the varied ratios of red and far-red light had significant effects on plant phenology. Far-red light creates a shading effect, giving plants the perception of reduced light. Consequently, plants respond by increasing height and leaf area, engaging in a competitive struggle for more light to ensure normal growth and enhance photosynthesis in the expanded leaf area (Hu et al., 2021; Mérai et al., 2023; Hu et al., 2024) The combination of red and far-red light can further regulate plant height, causing the above-ground portion to develop faster than the underground root system (Holalu et al., 2021). In our study, we observed that a red light to far-red light ratio of 3:2 significantly increased the dry/fresh weight of lettuce plants and promoted overall plant growth (**Figure 4**). This finding aligns with LI (Li and Kubota, 2009), who concluded that supplementing far-red light significantly enhances dry/fresh weight, leaf length, and leaf width in crops.

Various light qualities exert regulatory effects on physiological processes, including gas exchange and chlorophyll formation in plant leaves (Ramalho et al., 2002). The photoreceptors (such as phytochromes and cryptochromes) and chloroplasts within leaf cells play a role in regulating stomatal volume size and stomatal number in response to different light qualities. Notably, far-red light has a pronounced impact on the morphology of plant cells (Khattak and Pearson, 2006; Yonghua et al., 2005). In our experiment, it was observed that the addition of far-red light led to an increase in stomatal density (**Figure 7**). Stomata exhibited well-defined elliptical shapes, resulting in a significant increase in stomatal conductance and facilitated gas exchange. This finding aligns with previous studies that demonstrated an elevated far-red light ratio contributing to increased stomatal density in Chrysanthemum (Momokawa et al., 2011) and plants within the Chrysanthemum family (Kim et al., 2004).

The photosynthetic pigments in plant leaves play crucial roles in light energy absorption, transmission, and conversion, forming the foundation of photosynthesis. The composition and content of these pigments significantly influence the photosynthetic process (Lee et al., 2016). In our experiment, we observed a decrease in the content of photosynthetic pigments in lettuce leaves with the addition of far-red light. Notably, red light proved more favorable for the augmentation of chlorophyll b. Different light treatments had a significant impact on the pigment content of lettuce leaves. While there were no significant effects on chlorophyll a content, treatments F1, F2, and F3 increased the chlorophyll b content, with F4 showing the highest increase. Additionally, F1, F2, and F3 increased the total chlorophyll (a+b) content, with F1 being the most effective. The F3 treatment had the highest chlorophyll a/b ratio among all treatments. Although all light treatments increased carotenoid content compared to CK, only F1 and F3 showed significant differences (**Table 3**). The introduction of far-red light resulted in subsequent increases in the transpiration rate, intercellular CO_2_ concentration, and stomatal conductance. This response may be attributed to the pronounced shade avoidance effect induced by far-red light in lettuce. The plant perceives the shading of its leaves and senses reduced light, prompting an adaptive increase in transpiration and stomatal conductance (Naznin et al., 2019). Photosynthetic pigments serve as the material basis for photosynthesis and the foundation for nutrient synthesis. The supplementation of far-red light can influence quality by regulating the Red/Far-Red (R/FR) ratio. Far-red light treatment demonstrated an increase in the content of soluble sugars and soluble proteins in lettuce. This effect is likely due to the impact of varying R/FR values on the synthesis and absorption of carbohydrates and various amino acids in plants following increased far-red light exposure, consequently altering the content of soluble sugars and soluble proteins (Meng and Runkle, 2019).

The Fv/Fm ratio serves as an indicator of the efficiency of Photosystem II (PSII) in utilizing absorbed light energy to reduce the main quinone acceptor (QA) of PSII (Baker, 2008). Typically, an Fv/Fm value lower than 0.83 suggests plant stress, signifying a reduction in photosynthetic capacity (Björkman and Demmig, 1987). In this study, the Fv/Fm value exhibited a declining trend with the addition of far-red light. Notably, the F3 and F4 treatment groups recorded values below 0.83, However, it is important to note that ‘shade avoidance’ stress, which typically involves morphological adaptations such as stem elongation and does not directly influence Fv/Fm, may not be the correct terminology to describe our observations. Instead, the decline in Fv/Fm may be more likely associated with other stress factors such as high light intensity or environmental stresses (e.g., temperature, drought), which could exacerbate the production of reactive oxygen species (ROS) under these lighting conditions. In the F2 treatment, blue light was added, and the presence of blue light might alter the overall light quality balance perceived by the plants. This could affect the plants’ sensitivity to the increased ratio of far-red light, The observed changes in Fv/Fm and ΦPSII responses suggest an interaction of light quality with environmental factors, which could have been confounded by high plant density. Additionally, the signaling between blue light receptors and far-red light receptors (such as phytochrome) might interact, influencing the initiation of shade avoidance responses. (Forster and Bonser, 2009) ΦPSII represents the actual photosynthetic capacity of PSII, while ETR denotes the photosynthetic electron transfer rate. Our findings revealed a significant increase in ΦPSII with higher levels of red light. This increase is attributed to the enhanced activity of Photosystem I (PSI) induced by red light. Red light maximizes the absorption in chlorophylls, primarily benefiting PSII. Thus, a significant increase in ΦPSII might typically be expected with higher levels of red light rather than far-red light (**Table 4**). However, Zhen demonstrated that far-red light preferentially excites PSI over PSII, which can also increase ΦPSII. It is likely that under far-red light conditions, sufficient excitation of PSI helps balance the charges between PSII and PSI, leading to a reduced number of PSI centers. This reduction in PSI centers can limit the rate of electron transfer down the electron transport chain, causing PSII to relax slower than in other treatments. If the plants were dark-adapted for a longer period, the Fv/Fm values might be similar. The observed differences could also be influenced by high plant density and self-shading effects (Zhen et al., 2019).

Based on our principal component analysis (PCA) results, the study has significantly revealed the effects of different spectral treatments on plant growth and physiological characteristics (**Figure 11**). Specifically, the impacts of far-red light and red light on growth quality, biomass, photosynthetic indices, and chlorophyll fluorescence show distinct differences, providing an important basis for optimizing spectral treatments. The variable loading plot shows that chlorophyll a+b (BA), chlorophyll a (CA), and stem thickness (ST) exhibit strong positive loadings on PC1, indicating that these variables play a dominant role in distinguishing between far-red light and red light treatments. These results suggest that far-red and red light may regulate plant growth by affecting chlorophyll content and stem structure. Additionally, the F3 and F4 treatment groups are closely clustered in the sample score plot, indicating similarities in the considered variables. This finding provides a reference for optimizing light conditions in practical applications in the future. Overall, this study effectively highlights the different impacts of far-red and red light on plant growth and physiological characteristics through PCA, revealing the potential application value of spectral treatments in agriculture and horticulture. Future research can further explore the effects of different spectral combinations and intensities on various plant species and growth stages, aiming to achieve precise light environment control, thereby improving crop yield and quality.

## 6 Conclusion

In this study, we investigated the regulatory mechanism of far-red light on plant growth and physiology, specifically focusing on photosynthetic characteristics. Under the irradiation condition of the F3 treatment (with a red-to-far-red light ratio of 3:2), there was a significant increase in photosynthetic characteristics. Additionally, both stomatal conductance and quantity increased, resulting in enhanced gas exchange and improved light utilization and capacity in plants. The improved photosynthetic performance significantly enhanced the utilization of light energy by lettuce. This enhancement, in turn, promoted the growth, quality, and biomass accumulation of lettuce. The F4 treatment demonstrates promising application prospects. However, further adjustments in the red-to-far-red light ratio are necessary for optimal results. This research aims to provide a reference basis for the application of phosphor in horticultural plants.

## Data Availability

The datasets presented in this study can be found in online repositories. The names of the repository/repositories and accession number(s) can be found in the article/supplementary material.
